# Mesenchymal stem/stromal cells in the pathogenesis and regenerative therapy of inflammatory bowel diseases

**DOI:** 10.3389/fimmu.2022.952071

**Published:** 2022-08-04

**Authors:** Zhengping Che, Ziyu Ye, Xueying Zhang, Bihua Lin, Weiqing Yang, Yanfang Liang, Jincheng Zeng

**Affiliations:** ^1^ Dongguan Key Laboratory of Medical Bioactive Molecular Developmental and Translational Research, Guangdong Provincial Key Laboratory of Medical Molecular Diagnostics, Guangdong Medical University, Dongguan, China; ^2^ Department of Pathology, Dongguan Hospital Affiliated to Jinan University, Binhaiwan Central Hospital of Dongguan, Dongguan, China; ^3^ Guangdong Provincial Key Laboratory of Medical Molecular Diagnostics, School of Medical Technology, Guangdong Medical University, Dongguan, China; ^4^ Key Laboratory of Medical Bioactive Molecular Research for Department of Education of Guangdong Province, School of Basic Medicine, Guangdong Medical University, Dongguan, China; ^5^ Collaborative Innovation Center for Antitumor Active Substance Research and Development, Department of Biochemistry and Molecular Biology, School of Basic Medicine, Guangdong Medical University, Zhanjiang, China; ^6^ Dongguan Metabolite Analysis Engineering Technology Center of Cells for Medical Use, Guangdong Xinghai Institute of Cell, Dongguan, China

**Keywords:** inflammatory bowel diseases, ulcerative colitis, Crohn’s disease, mesenchymal stem/stromal cells, regenerative therapy

## Abstract

Inflammatory bowel diseases (IBDs) represent a group of chronic inflammatory disorders of the gastrointestinal (GI) tract including ulcerative colitis (UC), Crohn’s disease (CD), and unclassified IBDs. The pathogenesis of IBDs is related to genetic susceptibility, environmental factors, and dysbiosis that can lead to the dysfunction of immune responses and dysregulated homeostasis of local mucosal tissues characterized by severe inflammatory responses and tissue damage in GI tract. To date, extensive studies have indicated that IBDs cannot be completely cured and easy to relapse, thus prompting researchers to find novel and more effective therapeutics for this disease. Due to their potent multipotent differentiation and immunomodulatory capabilities, mesenchymal stem/stromal cells (MSCs) not only play an important role in regulating immune and tissue homeostasis but also display potent therapeutic effects on various inflammatory diseases, including IBDs, in both preclinical and clinical studies. In this review, we present a comprehensive overview on the pathological mechanisms, the currently available therapeutics, particularly, the potential application of MSCs-based regenerative therapy for IBDs.

## 1 Introduction

Inflammatory bowel diseases (IBDs) include two major types of disorders in gastrointestinal (GI) tract, ulcerative colitis (UC) and Crohn’s disease (CD) (**Table 1**), characterized by idiopathic gastrointestinal inflammation and tissue damage with a high recurrent rate (1). According to epidemiology, the prevalence of IBDs in Western countries is significantly higher than that in Eastern countries, but it is also rapidly increasing in Asian countries (2). The clinical signs and symptoms of the IBDs mainly include enteritis, diarrhea, recurrent hemorrhage, abdominal pain, reduced appetite, and weight loss, etc. (3, 4). Currently, there is still no cure for IBDs (5). In the early stages of IBDs, it is treated mainly with medication and surgery therapies, but the former can only control symptoms, while the latter is invasive and carries a high risk of complications (6). The clinical remission rates of IBDs range from 20% to 30% with monotherapy, but the remission rate would be around 50% if a combination of treatments were used (7). If the disease is not treated in time, the protracted course of IBDs can eventually trigger cancer, such as colitis-associated cancer (CAC), caused by external oncogenic factors (8, 9).

**Table 1:** The difference between Crohn’s and Ulcerative colitis.

Items/Type	Crohn’s (CD)	Ulcerative colitis (UC)
Causes	Inappropriate response of the immune system	Immune reaction, genetics
Risk factors	Smoking, environmental factors	Age, ethnicity
Lesion site	Anywhere between the mouth and anus	Rectum, colon
Symptoms	Abdominal cramping, diarrhoea, bloody stool, mucous stool, loss of appetite, weight loss, tiredness and mouth ulcers.	Diarrhoea, abdominal, anal pain, weight loss, tiredness, fatigue, rectal ulcers, bleeding, fevers, chills, anorexia and nausea
Complications	Nutritional deficiencies, fistulas, toxic, megacolon, narrowing of the intestines	Bleeding, toxic colitis, blood clotting, bowel cancer
Characteristics	Discontinuous lesions	Continuous lesions
Treatment	Lifestyle changes, medication and surgery	Self-care, medications and surgery
Medication	5-aminosalicylic acids, corticosteroids, immune system modulators, tumor necrosis factor-alpha antagonists, antibiotics, antidiarrhoeal medications	aminosalicylic acids, corticosteroids, biological therapies, antibiotics, probiotics and iron supplements
Surgery	It is used for fistulas, strictures (narrowing of the gut), large abscesses or other therapies have failed.	Medications is ineffective, precancerous or cancerous changes in the bowels, severe symptoms
Canceration	Low	High
Prognosis	Some people can be symptom-free for decades, while others may experience symptoms every few months.	There is a greater risk than normal of developing bowel cancer, usually after 7-10 years with ulcerative colitis.

**Figure 1:**
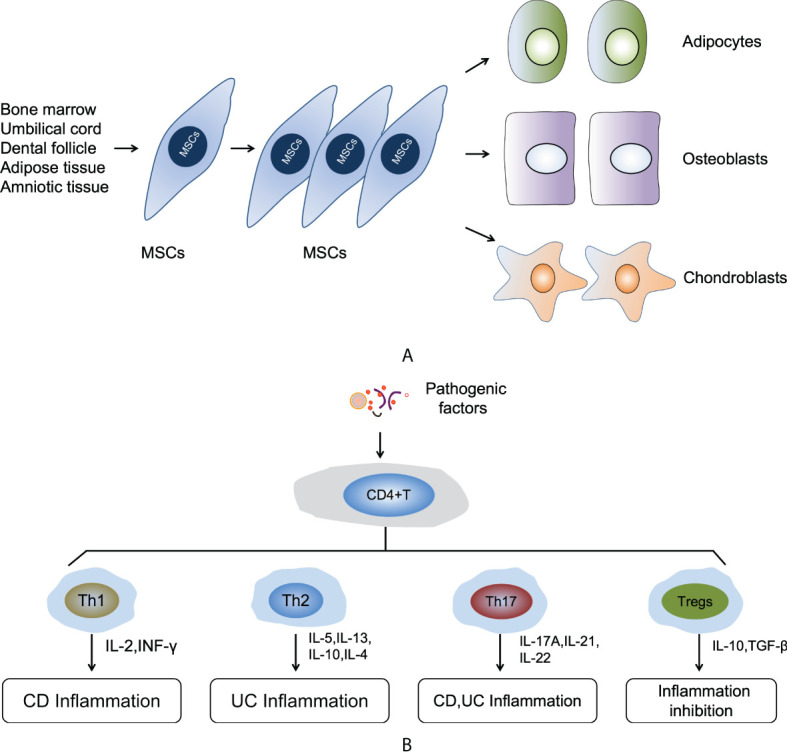
**(A)** The self-renewal and multipotent differentiation functions of MSCs. **(B)** The mechanism of immune responses in the pathogenic process of IBDs.

In recent years, mesenchymal stem cells (MSCs)-based therapy has emerged as a promising strategy for the treatment of IBDs due to their potent immuno-modulatory and tissue-repair functions (10). MSCs were first described by Friedenstein et al. as a population of bone marrow derived adherent cells, which were fibroblast-like and non-phagocytic cells but could differentiate into adipocytes, osteocytes, and chondrocytes under specific induction conditions *in vitro* (11). Bone marrow has long been regarded as the main source of MSCs. Nevertheless, isolation of bone marrow-derived MSCs (BM-MSCs) is a highly invasive process that may cause severe morbidity, while the number of BM-MSCs obtained usually decreases significantly with aging (12). In addition to bone marrow, MSCs have also been isolated from a variety of other tissues, such as adipose tissue (AT-MSCs), umbilical cord blood (UCB-MSCs), human amniotic tissue (HA-MSCs), and gingiva tissues (GMSCs) that are easily accessible (13). In order to standardize MSCs of different tissue origins, the International Society of Cell Therapy proposed three minimal standards for MSCs (1): plastic adherence *in vitro* culture conditions (2); the expression of a panel of phenotypic markers CD73^+^, CD90^+^, and CD105^+^ and the absence of hematopoietic marker CD11b^-^, CD14^-^, CD19^-^, CD34^-^, CD45^-^, CD79α^-^, and HLA-DR^-^ (3); the ability to differentiate into osteoblasts, adipocytes, and chondroblasts *in vitro* (14) (**Figure 1A**).

Given their wide existence in almost all tissues and organs of the body and their multifaceted biological functions, MSCs have been implicated to play essential roles in tissue homeostasis, regeneration, and diseases (15–19). Under physiological conditions, they may play an essential role in development, organogenesis, and maintenance of tissue and immune homeostasis through their cross-talks with specialized tissue cells and resident immune cells (15, 18, 19). On the other hand, in response to various insult signals, resident or endogenous MSCs may act as sensors of various insult signals and become activated by secreting a myriad of bioactive mediators that can foster or temper the immune/inflammatory responses, leading to the establishment of a pro-reparatory or regenerative microenvironment (20). However, various factors or pathological conditions, e. g. aging, can dysregulate the property and function of MSCs, thus contributing to the pathogenesis of various diseased conditions, including chronic inflammatory and fibrotic diseases (15–17, 21) and even tumorigenesis (20). In the review article, we also highlighted the latest research progress on the potential role of dysregulated MSCs in the pathogenesis of IBDs.

## 2 Pathogenic mechanism of IBDs

The etiology of IBDs is very complicated and has not yet been completely understood. To date, it is generally accepted that the pathogenesis of IBDs involves a complex interaction of environmental factors, genetic susceptibility, and dysregulated immune responses (22). Among the environmental factors, smoking, drugs use, diet habits, mental stress, and many other external factors are related to the occurrence of IBDs (23). In particular, smoking increases the risk of CD and is related with an increase in the recurrence rate of postoperative diseases (24). Air pollution can also increase the risk of CD and UC disease (25, 26). At the same time, Bitton et al. (27) also proposed that people with less stress would have less chance of developing IBDs. In addition, IBDs has a strong genetic tendency, especially in the first-degree relatives of patients who are at higher risk for IBDs. Compared with fraternal twins, identical twins have a higher prevalence rate of IBDs (28, 29). Genetic studies have reached a consistent conclusion: genetic factors play an important but non-decisive role in the occurrence of IBDs.

The dysregulated immune responses also play a key role in the pathogenic process of IBDs. The most fundamental pathogenetic patterns of IBDs is the dysregulation of innate and adaptive immunity. However, the adaptive immune responses are considered to be the main driver of IBDs (22). In response to different types of pathologic factors and inflammatory cytokines, naive CD4^+^ T cells can differentiate into distinct subsets of CD4^+^ T-helper (Th) cells, such as Th1, Th2, Th17, and CD4^+^FoxP3^+^ T regulatory cells (Tregs) (30). Th1 and Th17 cells can secrete a variety of inflammatory cytokines that induce intestinal epithelial inflammatory cells infiltrate and acute or chronic enteritis. However, intestinal inflammation can be suppressed *via* the differentiation Tregs and the supplementary of Th2 cells (31). Disturbance of immune homeostasis results in an imbalance of various subtypes of T cells as evidenced by increased proinflammatory cytokines derived from Th1 and Th17 cells in CD disease or Th2 cells in UC (32–34), and a decrease in the infiltration of anti-inflammatory Tregs in both CD and UC (35). For a long time, many studies believed that CD was driven by Th1 response, while UC was related to Th2 response. In the CD mucosa, macrophage-derived IL-12, IL-18, and TNF-α are overexpressed, driving the Th1 immune response to increase the production of IL-2 and IFN-ɤ. This response is thought to cause intestinal inflammation. In contrast, UC is characterized by increased expression of IL-5 and IL-13, which are members of the Th2 cytokine family, and this response will induce intestinal inflammation. In addition to the Th1 and Th2 responses, the role of Th17 cells, a subset of inflammatory T cells that expand under the action of pro-inflammatory cytokines, has been the focus research at this current stage Th17 cells are induced by IL-6 and TGF-β and produce IL-17A, IL-21, and IL-22, and those soluble factors will cause inflammation in CD and UC mucosa (36). IL-10 and TGF-β secreted by Tregs create an immunosuppressive microenvironment that is beneficial for repairing gastrointestinal dysfunction and colonic mucosal lesions (**Figure 1B**).

In recent years, accumulating evidence has highlighted the important role of resident MSCs in the establishment of a unique tissue niche that is essential for tissue and immune homeostasis, while the dysregulated MSCs might contribute to the development of various pathological conditions (15–17, 19, 21). Similarly, recent studies have also implicated the importance of intestinal MSCs in digestive organ development, mucosal tissue, and immune homeostasis, which can provide multiple niche signals to support functional integration of mucosal epithelial cells, immune cells, and gut microbiota (37–39). On the other hand, deficiency or aberrant activation of intestinal MSCs may lead to disturbance in mucosal and immune homeostasis, thus contributing to the pathogenesis of IBDs (40). Most recently, the emerging studies using scRNA-seq have significantly improved our understanding of the heterogeneity and the distinct role of diverse subsets of intestinal MSCs in regulating mucosal homeostasis and immunity by providing different niche signals under both physiological and inflammatory conditions (37, 38). For instance, Jasso et al. recently identified distinct subpopulations of stromal fibroblasts with gene signatures that are differentially regulated by chronic inflammation through scRNA-seq analysis of colon-derived mesenchymal stromal cells, thus providing mechanistic insight into how inflammation affects the function and behavior of intestinal MSCs and their crucial role in orchestrating mucosal tissue remodeling and healing (41).

## 3 Mechanisms of MSCs-based therapy of IBDs

### 3.1 MSCs-mediated immunomodulatory modulation

Compared with traditional therapeutics, MSC-based therapy is emerging as a promising platform for the treatment of IBDs. MSCs have the potential ability to restore immune homeostasis in patients with IBDs through turning the adverse pro-inflammatory mucosal immune responses into beneficial anti-inflammatory immune responses (42, 43). Many reported suggest that normal derived-MSCs (N-MSCs) can play a potential role in immunomodulatory or migrated to lesions to perform special functions during disease occurrence (44). However, the research on lesions-derived MSCs (L-MSCs) is still relatively fragmented (45, 46). L-MSCs, including but not limited to tumors, granulomas, oral, cervical, skin, precancerous, and white matter lesions, are the origin of disease occurrence and development (47–49). A few papers described the presence of L-MSCs possess many similarities with N-MSCs. It is noteworthy that L-MSCs may have directly or indirectly promoted the occurrence and development of local inflammatory diseases by increased proinflammatory factors and decreased anti-inflammatory factors (50). For instance, it has been reported that periapical lesions (PL)-MSCs possess similar immunomodulatory functions compared to N-MSCs (51). Dokic et al. reported that PL-MSCs increased the production of IL-6, IL-1β, TNF-α, and TGF-β, but not IL-8, thus exerting an pro-inflammatory role. These PL-MSCs can also inhibit T cell proliferation by suppressing IL-2 production and cell-cycle regulatory proteins. Additionally, TGF-β secreted by PL-MSCs can inhibit both Th1 and Th2 differentiation, and stimulate the expression of RORγt and FoxP3, the master regulators of Th17 and Tregs, respectively (52). Liu et al. (50) also showed that skin lesions-derived MSCs affects the activity of T lymphocytes in local lesions by increasing IL-11 secretion and reduced IL-6 and HGF. However, Galland et al. observed that tumor-associated MSCs (T-MSCs) have stronger immunosuppressive effect than N-MSCs, and affected both NK function and phenotype, such as the expression of CD56. T-MSCs shifted NK cells toward the CD56^dim^ phenotype and differentially regulate the function of a subset of CD56^bright/dim^. Moreover, T- and N-MSCs both affect degranulation and activating receptor expression in the CD56^dim^ subset, where they predominantly inhibit IFN-γ production to regulates immune function (53). Additionally, T-MSCs may largely rely on PGE2 and to a lesser extent on IL-6 to exert their immunosuppressive effects, and this effect by T-MSCs may be determined by signals derived from the tumor cells or the microenvironment, which may vary from patient to patient (20). Therefore, these studies have indicated that N- and L-MSCs possess immunomodulatory properties, which could make N- and L-MSCs based therapy of IBDs through mechanisms involving the secretion of anti-inflammatory soluble factors, direct cell-to-cell contact, and other regulatory pathways (54, 55).

#### 3.1.1 The secretion of anti-inflammatory soluble factors

The paracrine functions of transplanted MSCs are much complex and controversial under the pathological setting of IBDs. Upon injury, MSCs respond to insult signals and become activated by secreting an array of soluble bioactive factors that can serve as feedback signals to foster the immunomodulatory and tissue repair functions of MSCs (56). It is widely assumed that the property and function of MSCs are determined by the local microenvironment where they reside. Transplanted MSCs can secrete biological factors, e.g. anti-inflammatory or immunosuppressive factors, either “spontaneously” or following stimulation by pro-inflammatory cytokines or other soluble factors produced by local immune cells, such as IL-1β, TNF-α, and IFN-γ (57–59). For instance, Galland et al. demonstrated that T-MSCs exhibit an immunosuppressive phenotype mainly through PGE2 mediated suppression of NK cell function, including inhibition of IFN-γ production, the shift toward the CD56^dim^ phenotype, and downregulation of NK cell activating receptors (53). However, it has been reported that MSCs are not always weaponized with immunosuppressive functions. Fuenzalida et al. showed that MSCs pretreated with TLR3 ligands could secrete extra immunosuppressive cytokines and inhibit active T cells proliferation, while activation of TLR4 could prime MSCs to secrete proinflammatory factors (60, 61).

**Figure 2:**
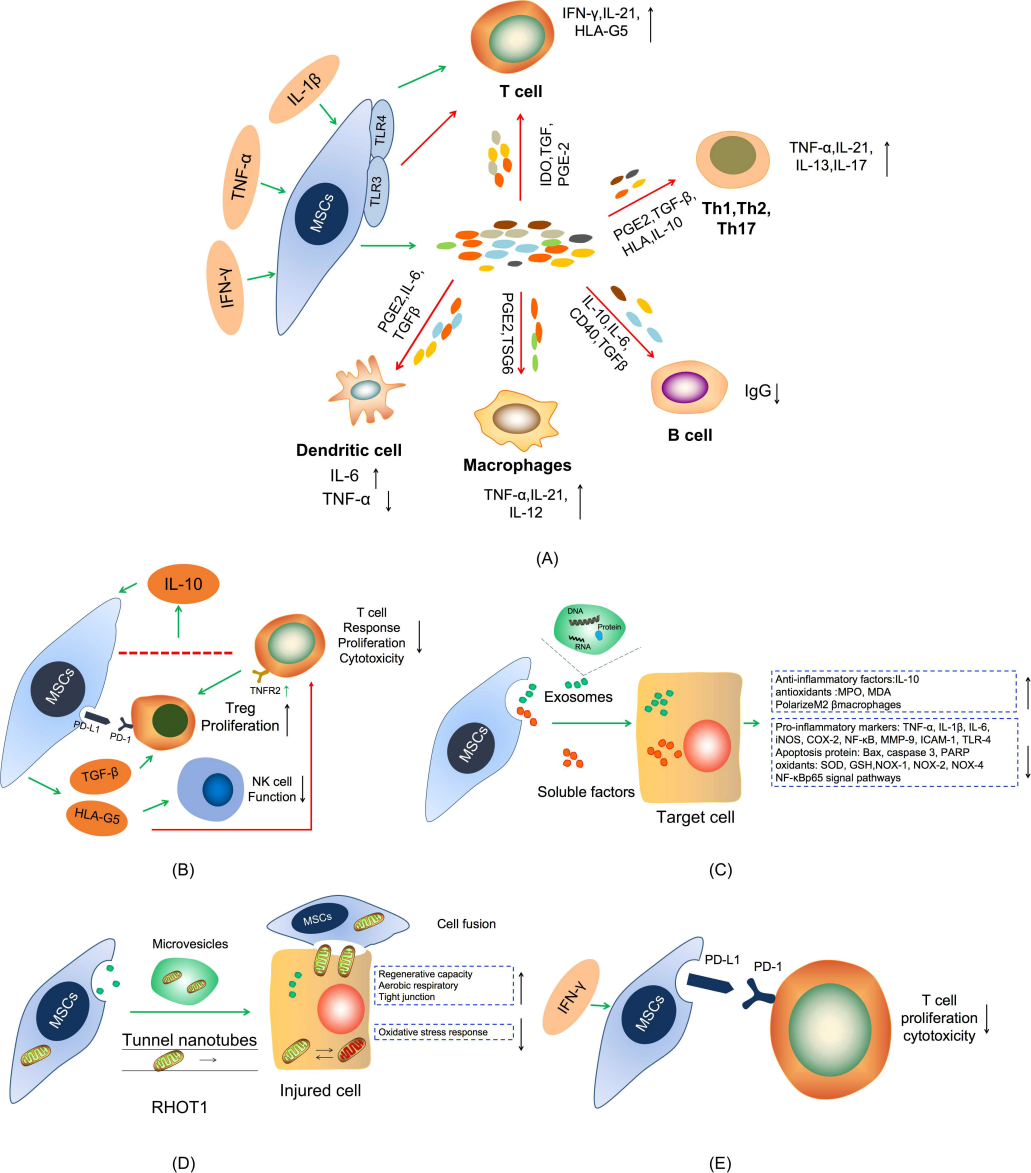
Schematic diagram of the mechanism of MSCs treatment in IBDs. **(A)** The secretion of anti-proliferative soluble factors. **(B)** Cell-To-Cell Contact. **(C)** Exosomes treatment. **(D)** Mitochondria transfer. **(E)** MSCs express PD-L1.

Under inflammatory conditions, transplanted MSCs stimulated by pro-inflammatory factors undergo a cascade of immune responses (62). Stimulated MSCs release PGE-2 and IL-6 to inhibit the maturation and function of dendritic cells (DCs), which leads to the decrease of TNF-α expression in myeloid DCs and upregulation of IL-1 level in lymphoid DCs (63, 64). MSCs inhibit T cell proliferation by elevating the expression of IDO and the secretion of TGF-β, IL-10, and PGE2, which can increase the expression of IFN- γ and IL- 21 (65). MSCs also could suppress macrophages activation through release of tumor-specific glycoproteins (TSGs) to convert the phenotype of macrophages from pro-inflammatory M1 macrophage characterized by secretion of inflammatory cytokines, e.g. TNF-α and IL-12, to anti-inflammatory M2 macrophages characterized by secretion of anti-inflammatory cytokines such as IL-10 and TGF-β (66). Naive CD4^+^ T cells differentiate into Th17 cells, which are characterized by the production of high levels of IL-17A, IL-17F, IL-21, and IL-22, upon combined stimulation of IL-6 and TGF-β, while their expansion is sustained by IL-23 secreted by macrophages and DCs (66, 67). It has been shown that MSCs restrain the development and activation of Th1 and Th17 cells by producing anti-proinflammatory factors, such as HLA, IL-10, TGF-β, and PGE2, and boost T cells and suppress B cells proliferation by promoting the expression of CD40, IL-6, IL-10, and TGF-β in colitis (68) (**Figure 2A**).

#### 3.1.2 Direct cell-to-cell contact mechanisms

In addition to secreting anti-inflammatory factors, research evidence shows that MSCs are also capable of enhancing IL-10 production through a cell-to-cell contact mechanism between activated T cells and MSCs, which in turn stimulates the release of soluble human leukocyte antigen G5 (HLA-G5) (69, 70). HLA-G5 can significantly downregulate naive and memory antigen-specific T cells response, inhibit T cell proliferation and cytotoxicity, and suppresses NK cell function. Meanwhile, MSCs can induce T cells to form functional Tregs through mechanisms involving TGF-β and PD-1/PDL-1 or depends on T cell-dependent expression of TNFR2 (71, 72). This mechanism of cell-to-cell contact reaction suggests that MSCs exert powerful immunosuppressive effects in IBDs (**Figure 2B**).

#### 3.1.3 MSCs-derived extracellular vesicles

Extracellular vesicles (EVs), including exosomes, are nanoscale microvesicles released by cells that play an important role in intercellular communication *via* transferring cargos containing various bioactive components (protein, DNA, mRNA, and non-coding RNA) involved in various of physiological and pathological processes (73, 74). There is increasing evidence that MSC-derived EVs display powerful therapeutic effects in several preclinical models of inflammatory diseases, suggesting that MSC-EVs may be a promising cell-free therapy because their properties are relatively stable and the safety risk is lower than that of the cell-producing similar products (75, 76). The mechanism of MSC-EVs in treating IBDs may be through inhibiting the secretion of pro-inflammatory cytokines and promoting the secretion of anti-inflammatory factors, regulating colonic macrophages, inhibiting apoptosis protein, regulating the expression of transcription factors, regulating signaling pathways, etc. (**Figure 2C**). For instance, Yang et al. found that intravenous injection of bone marrow mesenchymal stem cell-derived extracellular vesicles (BMSC-EVs) reduced the level of iNOS, COX-2, TNF-α, and IL-1β, inhibited apoptosis and NF- κBp65 signal transduction pathway, and regulated the balance between antioxidants (MPO, MDA) and oxidants (SOD, GSH) in IBDs mice (77). Liu et al. found that BMSC-EVs can act on colonic macrophages to produce IL-10, polarize intestinal M2β macrophages, inhibit inflammation and maintain the integrity of the colon barrier through metallothionein-2 (78). In addition to, Chang et al. found that adipose derived mesenchymal stem cell-derived extracellular vesicles (ADMSCs-EVs) effectively suppress acute inflammatory colitis in rats by down-regulating the expression of inflammatory markers (IL-1β, IL-6, TNF-α, NF-κB, COX-2, MMP-9, TLR-4, ICAM-1), oxidative stress markers (NOX-1, NOX-2, NOX-4) apoptotic, proteins (Bax, caspase 3, PARP), and fibrosis markers (Smad3, TGF-β) (79). Recently, a growing body of evidence has shown that EVs released by cells of the intestinal mucosa, immune cells, and gut microbiota play a significant role in maintaining the intestinal mucosal and immune homeostasis and the pathogenesis of IBDs (80). However, little is known about the role of EVs released by intestinal MSCs in both health and disease.

#### 3.1.4 MSCs-mediated mitochondria transfer

MSCs may exert their therapeutic effects through mitochondrial transfer, a process to transfer healthy mitochondria from MSCs to cells with mitochondrial dysfunction through the formation of tunnel nanotubes, gap junctions, microvesicles, cell fusion and separation, or other mechanisms to restore their aerobic respiratory function (81) (**Figure 2D**). MSCs are able to express high levels of RHOT1, a key RhO GTPase that supports mitochondrial transport from MSCs to adaptor cells (82). The transfer of mitochondria from MSCs with low levels of RHOT1 to injured intestinal epithelial cells is reduced in comparison with that from the MSCs with higher RHOT1. This reduction is not due to the decrease in nanotube formation, but to the decrease in RHOT1 mediated mitochondrial motility through the nanotubes. Mitochondria donated by MSCs can strengthen the tight junction of intestinal epithelial cells, provide sufficient ATP for intestinal epithelial cells, and reduce the oxidative stress response of recipient cells, thus relieving the intestinal symptoms of IBDs (83). In addition, MSCs can recognize mitochondria released from damaged intestinal epithelial cells as danger signals and activate the possibility of regenerative therapy (84). However, there is a need for further studies to determine the specific mechanism and key factors associated with mitochondrial transfer in MSC-based therapy for IBDs.

#### 3.1.5 Involvement of PD-L1 in MSCs-mediated immunosuppression

The regulation of immune checkpoint pathway plays pivotal roles in the treatment of immune system diseases (85). Among them, the programmed cell death-1 (PD-1)/programmed cell death-ligand 1 (PD-L1) checkpoint pathway is one of the important components to inhibit the immune response and maintain immune homeostasis (86). PD-1, a costimulatory molecule, is induced to be expressed on the surface of activated T cells, B cells and NK cells. PD-L1 (also known as B7 homolog 1, or B7-H1) is a ligand of PD-1. It is expressed in T cells, B cells, DCs, macrophages, and some non-hematopoietic cells. The PD-L1 binding with PD-1 prevents immune diseases by inhibiting the activity of T and B cells (87). However, recent studies have shown that MSCs also express PD-L1, PD-L2 that contribute independently to their immunosuppressive effects, which provides a new research direction for the treatment of IBD (88). In the inflammatory environment, the PD-L1 and PD-L2 induced by IFN-γ turned out to be constitutively expressed on MSCs derived from bone marrow, liver, and placenta. Meanwhile, PD-1 and PD-2 overexpression on the surface of T cells influences MSC-mediated inhibitory effects on T cell proliferation and cytotoxicity *in vitro* (89). Therefore, it is worthwhile to explore whether the expression of PD-L1 and PD-L2 can also contribute to MSC mediated immunosuppression in the treatment IBDs (**Figure 2E**).

#### 3.1.6 Signaling pathways

Several signaling pathways involved in immune and inflammatory responses have been implicated in MSC-based therapy of IBDs, including Wnt/β-catenin signaling pathway, the NF-κB pathway, the Notch signaling pathway, the PTEN/PI3K/Akt signaling pathway, and the FAS/FASL signaling pathway (90, 91). For instance, several reports have shown that MSCs inhibit the activity of macrophages, DCs, and T cells *via* TNF-α stimulated gene protein 6 (TSG-6) by activating the NF-κB signaling axis downstream of the CD44 receptor (92, 93). In addition, MSCs are capable of secreting monocyte chemotactic protein-1 (MCP-1) to promote apoptosis of CD4^+^ T cells *via* the FAS/FASL signaling pathway, thereby inhibiting T lymphocyte proliferation in IBDs (94).

### 3.2 MSCs alleviate IBDs *via* restoration of intestinal mucosal barrier

#### 3.2.1 Repair the intestinal microenvironment

The intestinal microenvironment has an important role in MSC-mediated therapeutic effects on IBDs. Under a normal environment, the sustainable renewal and proliferation of intestinal stem cells promote the continuous renewal of intestinal epithelium. During injury or damage to the intestinal tract, the disrupted microenvironment impairs the ability of intestinal stem cells to self-renewal, proliferate, and differentiate (95). The microenvironment of intestinal stem cells is jointly regulated by Wnt, Notch, and BMP signaling pathways (96). For instance, intestinal Paneth cells and pericryptal fibroblasts are essential components of the niche that mediate major signaling pathways of Wnt, Notch, and BMP to regulate the mechanism of self-renewal, proliferation, and differentiation of intestinal MSCs (97). Constituents of the crypt lumen produced by epithelial cells or bacteria may have potent effects on the intestinal stem cells. Furthermore, intestinal subepithelial myofibroblasts mediate interactions between epithelial and mesenchymal cells *via* secreting multiple morphogenetic factors involved in stem cell homeostasis. This process will determine the intestinal architecture and the balance between intestinal cell proliferation and differentiation (98).

#### 3.2.2 Repair intestinal epithelial cell

The view of “cell fusion” holds that when the *in situ* stem cells in tissues and mature cells are seriously damaged and unable to be rebuilt, the mature cells can be re-entered into the stem cell state by nuclear transfer (99). For example, BM-MSCs can differentiate into epithelial cells through “cell fusion” *in vitro* under certain culture conditions that include HGF, EGF, KGF, and IGF-II (100). In previous studies, Rizvi et al. used double-labeled intestinal epithelial cells with Y-FISH and enhanced green fluorescent protein immunohistochemical method, the former as a recipient source marker and the latter as a donor source marker. Among the tested cells, double-positive epithelial cells were found, which confirmed that donor BM-MSCs were involved in the repair of intestinal epithelial cell injury through the cell fusion mechanism after transplantation (101). However, Ferrand et al. argued that further studies are warranted to explore whether BM-MSCs can acquire epithelial characteristics through “cell fusion” with resident intestinal epithelial cells after engraftment (102).

#### 3.2.3 Tissue homing and tissue regeneration

The homing or recruitment of MSCs to the damaged tissue can also contribute an important role to the therapeutic efficacy of MSCs-based therapy for various inflammatory diseases, including IBDs. Tissue-oriented homing means that MSCs have the capacity to migrate and engraft specifically into damaged tissue sites, where they can differentiate into functional cells to replace damaged or diseased cells (103). Previous studies have shown that the molecular mechanisms underlying tissue homing of MSCs involve the expression of chemotactic receptors, matrix metalloproteinases (MMPs), and adhesion molecules. The chemotactic receptors mainly include CCR1, CCR2, CCR4, CCR5, CCR9, CXCR1, CXCR4, and CXCR5, whereby CCR2 and CCR4 assist the migration of MSCs, and CXCR4/SDF-1 axis can directly promote MSCs migration (104). The receptors expressed on MSCs can specifically bind to their ligands, which are released by certain histiocytes in the enteritis environment, such as CCL5, CCL19, CCL22, CCL25, CXCL8, CXCL13, etc. (105). MSCs may alleviate disease severity by expressing some adhesion molecules, such as CD29, CD44, CD49e, CD54, CD105, CD106, and CD166, which are essential for MSCs’ tissue homing (104). In addition, the adhesion molecules including P-selectin, VCAM-1, ALCAM, and VLA-4 have been demonstrated to promote the adhesion of MSCs to endothelial cells (106). Therefore, the expression of adhesion molecules may promote MSCs to integrate into damaged intestinal tissues to facilitate tissue regeneration through differentiation into intestinal epithelial cells, suppressing inflammation, and promoting angiogenesis (107). Additionally, several MMPs, such as MMP-2 and MT1- MMP, are also indispensable for tissue homing and tissue regeneration of MSCs. Tissue regeneration has been found after MSCs arrive at inflamed tissue (108). It is well accepted that MSCs contribute to tissue repair, mainly due to their ability to stimulate local tissue proliferation and survival by secreting proteolytic enzymes and angiogenic factors, while inhibiting tissue apoptosis and fibrosis (109). Some bioactive molecules, such as NO, IFN-γ, and TNF-α, can also stimulate tissue repair functions of MSCs through altering their migration, differentiation, or immunologic properties (110, 111). In particular, the migration rate and duration of MSCs are decisive factor affecting the efficiency of tissue repair and regeneration. However, there are still few studies on tissue homing and tissue regeneration through MSCs in the treatment of IBDs. **Table 2** Summarizes the related mechanisms of MSCs therapy.

**Table 2:** The mechanism of difference source of MSCs in treatment Inflammatory Bowel Disease.

MSCs source	*Model*	Type	Pathway	Dosage	Mechanism	Refs
BM-MSCs	C57BL/6 Mice	IBD	i.v.	1×10^6^ cells	Up-regulation of COX2 and the activation of EP4 receptors	Brown et al. (112)
BM-MSCs	C57BL/6 mice	UC	i.p.	2×10^6^ cells	Through suppression of DCs’ inflammatory phenotype through Gal-3	Nikolic et al. (113)
BM-MSCs	BALB/c mice	UC	i.p.	2×10^6^ cells	Promoted M2-like macrophage polarization and relieved inflammatory responses	Cao et al. (114)
BM-MSCs	Wister rats	UC	i.p.	2×10^6^ cells	By reducing the neutrophil infiltration, lipid peroxidation, and proinflammatory cytokine levels	Froushani et al. (115)
hUC-MSCs	Patients	IBD	i.v.	2.3-4.7×10^7^ cells	Accelerate the apoptosis of active inflammatory cells by down-regulating inflammatory mediator production	Hu et al. (116)
hUC-MSCs	KM mice	IBD	i.p.	1.3×10^6^ cells	By regulating the expression of IL-7	Fei et al. (117)
hUC-MSCs	BALB/c mice	IBD	i.p.	1×10^6^ cells	Modulation of immunosuppression by producing PGE2 inducing TLR3 to activate Notch-1 signaling	Qiu et al. (90)
hUC-MSCs	BALB/c mice	IBD	i.v.	n/a	Reduce ubiquitin-protein expression and reduction of NF-κB and mTOR activation	Wu et al. (118)
hUC-MSCs	C57BL/6 mice	IBD	i.p.	3×10^6^ cells	By inhibiting ERK signalling, polarize neutrophils toward the “N2” phenotype.	Wang et al. (119)
GMSCs	C57BL/6J mice	UC	i.v.	2×10^6^ cells	By downregulating the production of inflammatory cytokines by reducing colonic infiltration of inflammatory cells and promoting the generation/activation of Tregs	Zhang et al. (43)
GMSCs	C57BL/6J mice	UC	i.v.	n/a	By modulating inflammatory immune cells *via* IL-10 signalling	Lu et al. (120)
GMSCs	C57BL/6J mice	UC	i.v.	2×10^5^ cells	By upregulating expression of FAS ligand	Xu et al. (121)
GMSCs	C57BL/6J B6.129P2-Cbstm1Unc/J, and Cbs^+/−^ mice	UC	i.v.	2×10^5^ cells	By Fas/FasL coupling-induced T-cell apoptosis	Yang et al. (94)
GMSCs	C57BL/6J mice	UC	i.v.	1×10^6^ cells	By upregulating expression of FAS ligand	Yu et al. (122)
HA-MSCs	SD rats	IBD	i.v.	1×10^6^ cells	By producing a variety of humoral factors	Miyamoto et al. (123)
HA-MSCs	CD-1 mice	IBD	i.v.	2×10^6^ cells	By increasing the numbers of Lgr51 intestinal stem cells, stimulating intestinal epithelial cell proliferation, and increasing intestinal angiogenesis	Soontararak et al. (124)
AT-MSCs	C57BL/6J mice	IBD	i.p.	2×10^6^ cells	Increased release of TSG-6 and PGE2	Song et al. (125)
AT-MSCs	C57BL/6J mice	IBD	i.p.	1-5×10^6^ cells	Induces an innate immune memory response	Lopez-Santalla et al. (126)
AT-MSCs	SD rats	UC	i.v.	1×10^7^ cells	By suppressing NF-κB signaling pathway	Qi et al. (127)
MSC-CM	Rat	IBD	i.v.	4.5×10^7^ cells	Produced pleiotropic gut trophic factors	Watanabe et al. (128)
iPSC-MSCs	C57BL/6J mice	IBD	i.p.	2×10^6^ cells	Hyaluronan-CD44 interacts with TSG-6 in an Akt-dependent manner	Yang et al. (129)
DF-MSCs	CD patients	CD	i.v.	n/a	By inducing increased numbers of Tregs and reducing CD4^+^IL22BP T cell ratio	Zibandeh et al. (130)

## 4 Route of MSCs-based therapy for IBDs

The administration route can affect the therapeutic efficacy of MSCs for various pathological conditions, including IBDs. Two main approaches have been developed for administration of MSCs for IBDs treatment: local administration of MSCs for treatment of perianal fistulizing CD and systemic administration of MSCs for treatment of luminal inflammatory disease (131). Studies have demonstrated that local administration of autologous or allogeneic BM-MSCs and AT-MSCs achieved obvious clinical efficacy in patients with fistulazing CD by downregulating local immune responses and initiating wound healing (131). The effects of systemic administration of autologous or allogeneic MSCs have been evaluated in clinical trials., indicating that the systemic administration of AT-MSCs significantly improved the clinical outcome and prognosis for IBDs (132). However, there is still no standard reference for selecting the route for MSCs administration, either local or systemic administration, which might be determined according to the specification of diseases to be treated.

## 5 MSCs-based therapy for IBDs

MSCs can differentiate from other stem cells. Human pluripotent stem cells (hPSCs) include human embryonic stem cells (hESCs) and induced pluripotent stem cells (iPSCs), both of which can differentiate into MSCs (133). MSCs derived from hESCs and iPSCs exhibited similar properties, such as their ability to secrete anti-inflammatory soluble factors, and to restore the intestinal mucosal barrier in IBDs (134). hESCs-MSCs exhibit potent immunosuppressive on the colonic mucosa through preferentially homing to inflamed tissues and secondary lymphoid organs. It has a very effective inhibitory effect on the proliferation of Th1, but not the Th2 (135). However, iPSCs-MSCs can exhibit immunosuppressive directly inhibiting Th2 differentiation and promoting Tregs responses, depending on the mechanism of PGE2 production and cell-cell contact (136). For instance, Xu et al. reported that intravenous injection of hESCs-MSCs alleviated both acute and chronic DSS-induced colitis in mice through increasing endogenous IGF-1 secretion and maintaining colonic epithelial integrity and regenerative (137). Soontararak et al. showed that iPSCs-MSCs ameliorated clinical abnormalities in IBDs by stimulating intestinal epithelial cell proliferation increasing the numbers of Lgr5^+^ intestinal stem cells, and increasing intestinal angiogenesis, changing the microbiome in colitis and restoring its normal microecology (124).

Due to the self-renewal, multipotency, and immunosuppressive characteristics of MSCs, more clinical trials have been conducted to find a suitable treatment for IBDs using MSCs. In recent years, a growing number of clinical trials have proved the beneficial effects of MSCs on IBDs. Through searching the ClinicalTrials.gov database, 34 clinical trials have been identified on MSC based therapy for IBDs (**Table 3**). In these data, we found that different sources of MSCs, different injection methods, and doses of IBDs can improve clinical symptoms in different degrees. However, the clinical treatment of IBDs has a large sample size and a long cycle. The therapeutic effect still needs to be further observed.

**Table 3:** MSC-based clinical trials for inflammatory bowel disease.

ClinicalTrials.gov identifier	Status	Phase	Estimated Enrollment	Pathway	Dosage	Conditions	Type of cells	Country
NCT03299413	Active, not recruiting	Phase 1 Phase 2	20	i.v.	1.2×10^9^ cells	IBD	Wharton Jelly mesenchymal stem cells	Jordan
NCT03115749	Not yet recruiting	n/a	60	n/a	n/a	IBD	Intestinal mesenchymal stem stells	Montpellier
NCT01914887	Recruiting	Phase 1 Phase 2	8	Colonoscope	6×10^7^ cells	UC	Allogeneic adipose tissue-derived mesenchymal stem cells	Spain
NCT01874015	Recruiting	Phase 1	10	n/a	n/a	CD	Bone marrow mesenchymal stem cell	Spain
NCT01157650	Completed	Phase 1 Phase 2	15	n/a	n/a	CD	Autologous mesenchymal stem cells	United States
NCT00294112	Completed	Phase 2	10	i.v.	8×10^6^/2×10^6^ cells	CD	Adult human mesenchymal stem cells	United States
NCT02677350	Withdrawn	Phase 1	20	i.v.	2×10^7^ cells	CD	Allogeneic bone marrow derived human mesenchymal stem cells	United States
NCT02445547	Completed	Phase 1 Phase 2	82	i.v.	1×10^6^ cell/kg	CD	Umbilical cord mesenchymal stem cells	China
NCT00543374	Completed	Phase 3	98	i.v.	6×10^8^-1.2×10^9^ cells	CD	PROCHYMAL adult human mesenchymal stem cells	United States
NCT00482092	Completed	Phase 3	330	i.v.	6×10^8^-1.2×10^9^ cells	CD	Mesenchymal stem cells	United States
NCT01540292	Unknown status	Phase 1 Phase 2	20	i.v.	1.5-2.0 × 10^6^ cell/kg	CD	Mesenchymal stem cell	Belgium
NCT04519671	Recruiting	Phase 1 Phase 2	20	i.v.	7.5×10^7^ cells	CD	Bone marrow derived mesenchymal stem cells	United States
NCT04519684	Recruiting	Phase 1 Phase 2	20	i.v.	7.5×107 cells	CD	Bone marrow derived mesenchymal stem cells	United States
NCT01144962	Completed	Phase 1 Phase 2	21	Local injection	1×10^7^-9×10^7^ cells	CD	Bone marrow derived mesenchymal stem cells	United States
NCT04519697	Recruiting	Phase 1 Phase 2	20	Local injection	7.5×107 cells	CD	Mesenchymal stem cells	Netherlands
NCT04073472	Not yet recruiting	Phase 1	15	Local injection	6×10^7^ cells	CD	Bone marrow derived mesenchymal stem cells	United States
NCT04548583	Recruiting	Phase 1 Phase 2	24	Targeted endoscopic	1.5×10^8^-3×10^8^ cells	CD	Bone marrow derived mesenchymal stem cells	United States
NCT03183661	Enrolling by invitation	phase 1	9	i.v.	2×10^6^/8×10^6^ cell/kg	CD	Allogenic adipose-derived mesenchymal stem cells	China
NCT01221428	Unknown	Phase 1 Phase 2	50	i.v.	2×10^7^ cells	UC	Umbilical cord mesenchymal stem cells	Austria
NCT01541579	Completed	Phase 3	278	Local injection	1.2×10^8^ cells	CD	Adipose-derived stem cells	Austria
NCT04312113	Recruiting	Phase 1	20	Intra-arterial delivery	1.5×10^7^-3×10^7^ cells	UC	Autologous adipose-derived mesenchymal stem cells	United States
NCT04543994	Recruiting	Phase 1 Phase 2	24	Endoscopic delivery	1.5×10^8^/3×10^8^cells	UC	Bone marrow mesenchymal stem cell	United States
NCT01233960	Completed	Phase 3	73	Intra-arterial delivery	2×10^8^ cells	UC	Mesenchymal stem cells	United States
NCT03609905	Recruiting	Phase 1 Phase 2	50	Colonoscope	5×10^7^ cells	UC	Adipose-cord mesenchymal stromal cells	China
NCT03901235	Recruiting	Phase 1 Phase 2	60	Intratissular injection	n/a	n/a	Mesenchymal stem cells	Belgium
NCT02442037	Recruiting	Phase 1 Phase 2	30	i.v.	1×10^6^ cell/kg	UC	Umbilical cord derived mesenchymal stem cell	China
NCT02580617	Recruiting	Phase 1	9	n/a	1.5×10^7^-1×10^8^ cells	CD	Adipose-derived mesenchymal stem cells	Korea
NCT01510431	No longer available	n/a	n/a	n/a	2×10^8^ cells	CD	Mesenchymal stem cells	United States
NCT02403232	Recruiting	Phase 2	10	n/a	n/a	CD	Adipose tissue-derived stem cells	Italy
NCT02403232	Unknown status	Phase 1 Phase 2	24	i.v.	0.5×10^8^/1×10^8^ cells	CD	Umbilical cord blood derived-universal stem cells	Korea
NCT02926300	Recruiting	Phase 1 Phase 2	24	n/a	n/a	CD	Stem cells	Korea
NCT03220243	Completed	Phase 1	5	Local injection	2×10^7^ cells	CD	Mesenchymal stromal cell	United States
NCT01915927	Completed	Phase 1	20	Local injection	2×10^7^ cells	CD	Mesenchymal stromal cell	United States
NCT03449069	Recruiting	Phase 1	5	Local injection	2×107 cells	CD	Mesenchymal stromal cell	United States

n/a, not applicable.

Besides using MSCs to treat IBDs, it has also been shown that MSCs exhibit a powerful therapeutic function in CAC, which develops from chronic enteritis and frequently occurs in areas of chronic inflammation (31). The research found that hUC-MSCs migrated into the intestinal structure and then moved to the colon to reduce the number of tumors with the reduction of Ki67 by inhibiting chronic inflammation and the Smad2 signaling pathway (138). However, the therapeutic effect of MSCs in colorectal cancer is in dispute due to the potent immunosuppressive properties of MSCs that can contribute to the immunosuppressive tumor microenvironment favoring immune evasion of cancer cells, and thus, negatively affect the therapeutic effect of CAC (139).

MSCs-based therapy for many other diseases has also shown great promise. Numerous basic studies and clinical trials have proved that MSCs exhibit an obvious therapeutic effect on nervous system diseases, cardiovascular system diseases, pulmonary lung diseases, etc., and show potent regenerative potential in diseased liver, lung, kidney, skin, and other organs (140, 141). The regenerative therapeutic potentials of MSCs are mainly attributed to their unique properties, such as self-renewal and multipotent differentiation capability, immunomodulatory/anti-inflammatory function, easy isolation and expansion *in vitro*, etc. Meanwhile, the lack of expression of the major MHC II molecule and the low expression level of MHC I and co-stimulatory molecule (CD40, CD80, CD86, and CD154) coin MSCs with a hypoimmunogenic and immune tolerant phenotype, which allows MSCs to escape immune recognition and clearance *in vivo* delivery (142). In addition, MSCs have great application value in tissue engineering, wound repair, gene therapy, cell replacement therapy, etc (143).

## 6 Unanswered questions and future perspectives

Even though the regenerative and therapeutic potentials of MSCs have been widely studied in both preclinical studies and clinical trials, it remains largely unknown about the cellular and molecular mechanisms underlying MSC mediated therapeutic effects *in vivo*. There are still numerous issues to be solved when MSCs are widely used in the clinic, such as the suitable source of MSCs, the dosage and modality of administration, the long-term fate of transplanted cells, and the potential side effects e.g. tumorigenicity following transplantation, etc. Similarly, such issues also exist in MSC-based therapy of IBDs. Therefore, more in-depth mechanistic basic and preclinical studies, clinical trials, and long-term follow-up are required to establish optimal treatment modalities for MSC based therapy of IBDs.

### 6.1 Sources of MSCs

Sources of MSCs will obviously influence the therapeutic effect. For a long time, BM-MSCs are the main source for the acquisition of MSCs, but their isolation is an invasive treatment method (12). Therefore, alternative sources of MSCs, such as umbilical cord blood and adipose tissue, have been aggressively pursued. The biggest advantages of AT-MSCs are that they can be acquired in large numbers and are less invasive procedures. There is a growing body of data showing differences between BM-, AT-, and UCB-MSCs, including their immunomodulatory properties (144). Several reports found that AT- and UCB-MSCs may suppress immune responses more effectively than BM-MSCs *in vitro* (145). However, there are still few studies on the sources of L-MSC in the treatment of IBDs. Therefore, studies comparing their efficacy *in vivo* will need to be done to choose the best type of MSCs to use for IBDs treatment.

### 6.2 Modalities and dosage of administration

There is a general agreement by now that local injection of MSCs is the most suitable route of administration for treating IBDs. In the recent trials, data have shown that local injection of MSCs into the fistula wall itself was appropriate for perianal fistulas, which makes partial healing of the lesion without rejection of the cells and adverse effects. The detailed procedure of the local injection has a significant impact on the observation and needs to be better elucidated (146). Conversely, systemic administration is a better option in luminal CD disease. Because the intravenous injection is easy, minimally invasive, and safe for patients and plays an important role in the attenuation or progression of CD (147). However, accumulating evidence has shown that a small part of MSCs through intravenous injection is easily stuck in the lungs, so the proportion of MSCs reaching the inflamed intestine needs to be further evaluated. Meanwhile, it is vital that the amount of MSCs transplanted to patients is clearly defined, with an eye toward balancing safety with efficacy in MSC-based therapy (148). The dose of MSCs administration were determined by the sources of MSCs, injection method, and the type of disease. A large number of experiments show that lower dose of MSCs seems to have a higher healing rate in perianal fistulizing Crohn’s disease. According to Molendijk et al’s experiments, the higher healing rate was observed in patients that received 3×10^7^ MSCs when compared to patients that received 9×10^7^ MSCs (131). Therefore, the optimal number of MSCs cannot be determined due to the impact of multiple factors, and dose escalation study is required to address this problem under the condition of the same independent variable.

### 6.3 Combination of MSCs and immunosuppressant

The combination of MSCs and other drugs will alter the therapeutic effect. MSCs have been used together with immunosuppressive drugs due to their shared common targets in clinical studies. As reported by Duijvestein M, incubation of MSCs with physiological concentrations of immunosuppressive drugs, such as azathioprine, mercaptopurine, methotrexate, and anti-TNFα compounds, does not directly alter the phenotypical, functional properties, survival, and inhibitory effects on peripheral blood mononuclear cell growth *in vitro*. There may even be an additive effect between 6-mercaptopurine and anti-TNFα antibodies (149). However, azathioprine can reduce the proliferation of rat BM-MSCs and increase their apoptosis and necrosis at a higher level *in vitro* (150). Dexamethasone has been shown to restrain the expression of iNOS and IDO, thus reversing MSC-mediated immunosuppression *in vitro* and abolishing the therapeutic effects of MSCs *in vivo* (151). The clinic experiments have also proven that steroids and MSCs should not be administered in combination (152). Nevertheless, there are few studies on the use of L-MSCs in combination with immunosuppressants in IBDs treatment. Therefore, the molecular mechanisms of interaction between MSCs and immunosuppressants should be further studied carefully in order to enable more effective manipulation of MSCs function for clinical applications.

### 6.4 MSC-related adverse event

The research showed that intravenous MSCs may cause mild and transient fever, headache, insomnia, dysgeusia, and diarrhea, but these symptoms will disappear after a period of time (153). To date, no serious MSCs related adverse effects have been reported in clinical studies, including clinical trials with patients suffering from IBDs or other diseases (154). Nevertheless, the most worrying adverse effect was whether MSCs have the potential to promote tumor growth and mitigate the effectiveness of treating enteritis due to their tumorigenic characteristics activated by oncogenes (155). To date, the clinical adverse effects have not been fully understood and then further studies are warranted in future clinical studies.

## 7 Conclusion

MSCs based therapy has unique advantages and shows its irreplaceable potential in medical applications nowadays. It represents a novel therapeutic option for IBDs and other diseases, showing durable efficacy, low trauma, and low recurrence rates, even in cases in which healing cannot be achieved with biologics or conventional surgical procedures cannot be performed. However, MSCs based therapy for IBDs is still at an exploratory stage and further basic mechanistic and clinical studies are warranted.

## Author contributions

All authors contributed significantly to the drafting and editing of this manuscript. JZ, ZC, and WY conceived the manuscript idea and wrote the manuscript. YL, WY, and JZ revised the manuscript content. ZY, XZ, and BL created the manuscript tables and figures. All authors contributed to the article and approved the submitted version.

## Funding

This study was supported by grants from the Dongguan Social Science and Technology Development Project (20211800904532, 201950715025192), Natural Science Foundation of Guangdong Province (2021B1515140004, 2021B1515140066, 2019A1515110042, 2019A1515011713), Characteristic Innovation Experimental Project of Ordinary Universities in Guangdong Province (2020KTSCX044), Discipline Construction Project of Guangdong Medical University, Research Foundation of Guangdong Medical University for Ph.D. Staff (GDMUB2019038, GDMUB2020017), the Medical Science Foundation of Guangdong Province (A2021438, A2020211).

## Acknowledgments

This study was supported by Dongguan Key Laboratory of Medical Bioactive Molecular Developmental and Translational Research.

## Conflict of interest

The authors declare that the research was conducted in the absence of any commercial or financial relationships that could be construed as a potential conflict of interest.

## Publisher’s note

All claims expressed in this article are solely those of the authors and do not necessarily represent those of their affiliated organizations, or those of the publisher, the editors and the reviewers. Any product that may be evaluated in this article, or claim that may be made by its manufacturer, is not guaranteed or endorsed by the publisher.
